# Prognosis of mechanically ventilated patients entering the weaning process

**DOI:** 10.1186/2197-425X-3-S1-A426

**Published:** 2015-10-01

**Authors:** G Beduneau, T Pham, JCM Richard, F Schortgen, JM Chretien, A Mercat, J Mancebo, L Brochard, WIND Study Group on behalf of REVA Network

**Affiliations:** University Hospital, Rouen, France; Medical and Surgical Intensive Care, Hôpital Tenon, APHP, Paris, France; Annecy Genevois General Hospital, Annecy, France; Medical ICU, CHU Henri Mondor, APHP Paris, Paris, France; Clinical Research Institute Angers Universitary Hospital, Angers, France; Medical Intensive Care, Universitary Hospital Angers, Angers, France; Hospital de Sant Pau, Barcelona, Spain, Barcelona, Spain; Saint Michael's Hospital and Keenan Research Centre, Interdepartmental Division of Critical Care, University of Toronto, Toronto, Canada

## Introduction

The WIND study “Weaning according to New Definition " prospectively collected epidemiologic data concerning mechanical ventilation (MV) and weaning. The aim of the present analysis was to specifically address mortality at the different pre-defined steps of the weaning process.

## Methods

This is a subsequent analysis of the original prospective observational study run in 36 intensive care units in France, Spain and Switzerland over a three month period. All patients requiring intubation and MV were enrolled. MV modality, results of spontaneous breathing trials (SBT) and extubation, therapeutic limitation as well as survival were daily collected until ICU discharge or day 60. A weaning attempt (WA) was defined either by a spontaneous breathing trial or a direct extubation (planned or unplanned). Weaning success was defined as discharged alive without mechanical ventilation within the 7 days following extubation. The prognosis of patients at the key steps of the weaning process was analysed as mortality at ICU discharge or D60.

## Results

2729 patients were enrolled and 20 specifically intubated for upper airway obstacle were excluded. 2709 patients were analysed among whom 2051 entered the weaning process (i.e. had at least one WA). Prognosis is reported on table 1 for the whole cohort, for the patients entering weaning, after failure of the first WA and for the patients still ventilated 7 days after the first WA.

**Table 1:** After failure of 1^st^ WA, 125 patients (among the 197 dead) died in the seven first days.

	Intubated patients	Entering weaning (WA)	Failure 1st WA	Still ventilated 7 days after 1st WA
Patients (N)	2709	2051	605	218
Mortality, % (N)	28 (771)	10 (205)	33 (197)	28 (62)

## Conclusions

Based on this large cohort of intubated patients, mortality was 10% for the patients entering the weaning process but was three times higher (33%) in patients failing their 1^st^ WA; 63% of the deaths occurred within one week after the failed WA. The present analysis is the first to document mortality along different steps of the weaning process as experienced in daily practice.

## Grant Acknowledgment

Association d’aide aux insuffisants respiratoires (Rouen, France)

*GB and TP are considered both as first authors.

